# Artificial intelligence to guide rituximab therapy in patients with phospholipase A2 receptor–associated membranous nephropathy

**DOI:** 10.1093/ckj/sfaf113

**Published:** 2025-04-16

**Authors:** Maxime Teisseyre, Alexandre Destere, Marion Cremoni, Sonia Boyer-Suavet, Alexandre Karamé, Léa Corlu, Gabrielle Duneau, Paule Poirier, Siriane Lefevre, Kévin Zorzi, Céline Fernandez, Vesna Brglez, Sylvia Benzaken, Guillaume Favre, Milou-Daniel Drici, Vincent L M Esnault, Sylvain Benito, Barbara Seitz-Polski

**Affiliations:** Centre de Référence Maladies Rares Syndrome Néphrotique Idiopathique et Glomérulonéphrite Extra-membraneuse, CHU de Nice, Université Côte d'Azur, Nice, France; Département de Néphrologie, Dialyse et Transplantation, CHU de Nice, Université Côte d'Azur, Nice, France; Institut de Recherche sur le Cancer et Vieillissement UMR7284 CNRS INSERM U1081, Université Côte d'Azur, Nice, France; Laboratoire d'Immunologie, CHU de Nice, Université Côte d'Azur, Nice, France; Département de Pharmacologie Clinique et de Pharmacovigilance, CHU de Nice, Université Côte d'Azur, Nice, France; Université Côte d'Azur, Inria, CNRS, Laboratoire J.A. Dieudonné, Maasai team, Nice, France; Centre de Référence Maladies Rares Syndrome Néphrotique Idiopathique et Glomérulonéphrite Extra-membraneuse, CHU de Nice, Université Côte d'Azur, Nice, France; Département de Néphrologie, Dialyse et Transplantation, CHU de Nice, Université Côte d'Azur, Nice, France; Institut de Recherche sur le Cancer et Vieillissement UMR7284 CNRS INSERM U1081, Université Côte d'Azur, Nice, France; Laboratoire d'Immunologie, CHU de Nice, Université Côte d'Azur, Nice, France; Département de Néphrologie et Dialyse, Centre Hospitalier d'Antibes Juan-Les-Pins, Antibes, France; Département de Néphrologie et Dialyse, Néphropôle – Médipole Hôpital Privé, Lyon Villeurbanne, Villeurbanne, France; Département de Néphrologie et Dialyse, Centre Hospitalier de Lorient, Lorient, France; Fondation AUB santé Lorient, Lorient, France; Département de Néphrologie et Dialyse, Centre Hospitalier d'Annecy Genevois, Annecy, France; Département de Néphrologie et Dialyse, Centre Hospitalier de Lorient, Lorient, France; Centre de Référence Maladies Rares Syndrome Néphrotique Idiopathique et Glomérulonéphrite Extra-membraneuse, CHU de Nice, Université Côte d'Azur, Nice, France; Département de Néphrologie, Dialyse et Transplantation, CHU de Nice, Université Côte d'Azur, Nice, France; Institut de Recherche sur le Cancer et Vieillissement UMR7284 CNRS INSERM U1081, Université Côte d'Azur, Nice, France; Centre de Référence Maladies Rares Syndrome Néphrotique Idiopathique et Glomérulonéphrite Extra-membraneuse, CHU de Nice, Université Côte d'Azur, Nice, France; Institut de Recherche sur le Cancer et Vieillissement UMR7284 CNRS INSERM U1081, Université Côte d'Azur, Nice, France; Centre de Référence Maladies Rares Syndrome Néphrotique Idiopathique et Glomérulonéphrite Extra-membraneuse, CHU de Nice, Université Côte d'Azur, Nice, France; Institut de Recherche sur le Cancer et Vieillissement UMR7284 CNRS INSERM U1081, Université Côte d'Azur, Nice, France; Laboratoire d'Immunologie, CHU de Nice, Université Côte d'Azur, Nice, France; Laboratoire d'Immunologie, CHU de Nice, Université Côte d'Azur, Nice, France; Département de Néphrologie, Dialyse et Transplantation, CHU de Nice, Université Côte d'Azur, Nice, France; Département de Pharmacologie Clinique et de Pharmacovigilance, CHU de Nice, Université Côte d'Azur, Nice, France; Centre de Référence Maladies Rares Syndrome Néphrotique Idiopathique et Glomérulonéphrite Extra-membraneuse, CHU de Nice, Université Côte d'Azur, Nice, France; Département de Néphrologie, Dialyse et Transplantation, CHU de Nice, Université Côte d'Azur, Nice, France; EXACTCURE, Nice, France; Centre de Référence Maladies Rares Syndrome Néphrotique Idiopathique et Glomérulonéphrite Extra-membraneuse, CHU de Nice, Université Côte d'Azur, Nice, France; Département de Néphrologie, Dialyse et Transplantation, CHU de Nice, Université Côte d'Azur, Nice, France; Institut de Recherche sur le Cancer et Vieillissement UMR7284 CNRS INSERM U1081, Université Côte d'Azur, Nice, France; Laboratoire d'Immunologie, CHU de Nice, Université Côte d'Azur, Nice, France

To the Editor,

Rituximab is a first-line treatment for membranous nephropathy (MN) with proven safety and efficacy. Its bioavailability is reduced in nephrotic patients as compared with other rituximab-treated diseases without proteinuria [1]. Rituximab underdosing (serum level <2 µg/mL at Month 3) is a risk factor for treatment failure [1]. Optimal rituximab regimen remains uncertain. A high-dose rituximab regimen (two 1000 mg infusions given 2 weeks apart) proved more effective than the GEMRITUX regimen (two 375 mg/m^2^ infusions 1 week apart) in depleting B cells, anti-phospholipase A2 receptor (PLA2R) antibodies and inducing clinical remission [2]. Serum rituximab levels were higher at Month 3 with the high-dose regimen, which may explain the better outcome [2]. We have previously developed a machine-learning algorithm to predict the risk of rituximab underdosing in MN patients [3]. Here, we prospectively evaluate the efficacy and safety of an algorithm-guided rituximab regimen. Fourteen patients with PLA2R-associated MN were followed in the nephrology departments of Annecy, Antibes, Lorient, Villeurbanne and Nice (France) (Table 1). The rituximab dose was validated based on the risk of underdosing assessed using our algorithm [3]. Seven patients (50%) had a risk of underdosing estimated by our algorithm to be <50% and received a cumulative dose of 2000 mg of rituximab in two infusions (Days 0 and 15). Three patients (21%) had an estimated risk of underdosing of 50%–75% and received a cumulative dose of 3000 mg of rituximab in three infusions (Days 0, 15 and 30). Finally, four patients (29%) had an estimated risk of underdosing >75% and received a cumulative dose of 4000 mg of rituximab in four infusions (Days 0, 15, 30 and 45). Three of these last four patients were previously considered as refractory to rituximab. Proteinuria, serum albumin, anti-PLA2R antibody titer and glomerular filtration rate improved significantly during follow-up (Supplementary data, Fig. S1). At Month 3, five patients (36%) achieved clinical remission, one complete and four partial. All patients but one achieved immunological remission at Month 3. At Month 6, 12 patients (86%) achieved immunological and clinical remission, 1 complete and 11 partial. Immunological remission was achieved at Month 3 and partial clinical remission at Month 6 in the three patients previously considered refractory to rituximab. These results are superior to those published in the major international prospective studies (Supplementary data, Table S1). The treatment was well tolerated and no serious adverse drug reactions were reported. Measurement of residual rituximab levels at Month 3 shows that there is no risk of overdosing with algorithm-guided treatment (Table 1). Only one patient developed anti-rituximab antibodies. We then compared the clinical remission rate at Month 6 in our prospective cohort receiving algorithm-guided rituximab treatment with that in a retrospective cohort of 34 patients with PLA2R-associated MN receiving standard rituximab treatment (Table 1). The rate of clinical remission at Month 6 according to the risk of underdosing was significantly higher with algorithm-based treatment than with standard treatment (*P *= .02; Supplementary data, Fig. S2).

**Table 1:** Patients’ characteristics and outcome.

Variables	Patients treated prospectively with personalized rituximab protocol (*n* = 14)	Patients treated retrospectively with standard rituximab protocol (*n* = 34)	*P*-value
Characteristics at 1st rituximab infusion			
Age (years)	60 (53–69)	58 (42–69)	0.7
Sex (male/female)	11/3	24/10	0.7
Weight (kg)	75.9 (67.4–96.6)	75.2 (66.4–89.5)	0.5
Height (cm)	172 (169–181)	172 (165–179)	0.5
Body mass index (kg/m^2^)	26.8 (22.7–29.1)	26.1 (23.5–28.2)	0.6
Serum albumin (g/L)	25.1 (12.7–31.5)	22.8 (17.2–28.4)	0.8
Urine protein–creatinine ratio (g/g)	6.8 (5.6–9.4)	5.6 (4.1–11.3)	0.4
Serum creatinine (µmol/L)	134 (105–209)	128 (96–189)	0.5
Anti-PLA2R titer (RU/mL)	60 (48–207)	157 (68–337)	0.1
PLA2R epitope spreading, *n* (%)	9 (64)	23 (68)	1
Previous rituximab treatment, *n* (%)	11 (79)	2 (6)	<0.0001
Last rituximab treatment (months)	26 (6–46)	32 (9–54)	0.8
Previous cumulative dose of rituximab (mg)	2000 (2000–6000)	2000 (2000–2000)	0.9
Current dose of rituximab, *n* (%)			
1000 mg D0-D15	7 (50)	34 (100)	
1000 mg D0-D15-D30	3 (21)	0 (0)	
1000 mg D0-D15-D30-D45	4 (29)^a^	0 (0)	
Supportive therapy, *n* (%)	14 (100)	34 (100)	
Outcome at Month 3^b^			
B cell depletion (yes/no)^c^, *n*/*n* (%)	10/0 (100)	18/6 (75)	0.1
Serum rituximab level (µg/mL) ^d^	4.6 (2.9–9.1)	0.6 (0.0–7.7)	0.2
Clinical remission, *n* (%)	5 (36)	14 (41)	1
Immunological remission, *n* (%)	13 (93)	20 (59)	0.04
Outcome at Month 6^b^, *n* (%)			
Clinical remission	12 (86)	15 (44)	0.01
Immunological remission	12 (86)	21 (62)	0.2

Categorical data are described as frequencies and percentages and were compared using a chi-squared test or Fisher's exact test. Quantitative data are described as median and interquartile range and were compared using the Wilcoxon–Mann–Whitney test.

a One of these patients was also receiving cyclosporine.

b Complete remission was defined as a UPCR <0.3 g/g, accompanied by a normal serum albumin concentration. Partial remission was defined as a UPCR <3.5 g/g with a >50% reduction compared with baseline value accompanied by an improvement of the serum albumin concentration. Immunological remission was defined by an anti-PLA2R antibody titer <14 RU/mL by enzyme-linked immunosorbent assay (EUROIMMUN). B-cell depletion was defined as a CD19^+^ cell count <5/mm^3^.

c CD19^+^ counts were measured at Month 3 in 10 patients (71%) from the prospective cohort and 24 patients (71%) from the retrospective cohort.

d Serum rituximab levels were measured at Month 3 in nine patients (64%) from the prospective cohort and 33 patients (97%) from the retrospective cohort.

D0, Day 0; D15, Day 15; D30, Day 30; D45, Day 45; UPCR, urine protein–creatinine ratio.

To conclude, our algorithm provides an early estimate of the risk of rituximab underdosing, allowing the initial dose to be tailored to the patient leading to faster remission. This personalized rituximab regimen appears to be effective in previously rituximab-refractory patients, avoiding the need to intensify treatment with cyclophosphamide and corticosteroids, which is associated with frequent and serious side effects [4]. However, the number of subjects in our pilot study is limited. Further studies are needed to draw robust conclusions. The contribution of our algorithm to daily practice in primary MN (PLA2R-associated or not) will be evaluated in a randomized controlled trial (NCT06341205).

## Supplementary Material

sfaf113_Supplemental_File

## References

[1] Teisseyre M, Cremoni M, Boyer-Suavet S. et al. Rituximab immunomonitoring predicts remission in membranous nephropathy. Front Immunol 2021;12:738788. 10.3389/fimmu.2021.738788 34721403 PMC8548826

[2] Seitz-Polski B, Dahan K, Debiec H. et al. High-dose rituximab and early remission in PLA2R1-related membranous nephropathy. Clin J Am Soc Nephrol 2019;14:1173–82. 10.2215/CJN.11791018 31340979 PMC6682825

[3] Destere A, Teisseyre M, Merino D. et al. Optimization of rituximab therapy in adult patients with PLA2R1-associated membranous nephropathy with artificial intelligence. Kidney Int Rep 2024;9:134–44. 10.1016/j.ekir.2023.10.023 38312797 PMC10831377

[4] van den Brand JAJG, Ruggenenti P, Chianca A. et al. Safety of rituximab compared with steroids and cyclophosphamide for idiopathic membranous nephropathy. J Am Soc Nephrol 2017;28:2729–37. 10.1681/ASN.2016091022 28487395 PMC5576929

