# Supplementary material for: Artificial intelligence to guide rituximab therapy in patients with phospholipase A2 receptor–associated membranous nephropathy
Source: Clin Kidney J. 2025 Apr 16;18(5):sfaf113. doi: 10.1093/ckj/sfaf113 (PMC12046509; doi:10.1093/ckj/sfaf113)
Supplement: sfaf113_Supplemental_File [file sfaf113_supplemental_file.docx]

**SUPPLEMENTARY MATERIAL**

**Supplementary Figure 1. Trends in urine protein-creatinine ratio (A); serum albumin (B); anti-PLA2R antibody titer (C); and estimated glomerular filtration rate (D), between baseline and 6 months after algorithm-guided rituximab treatment.**

**
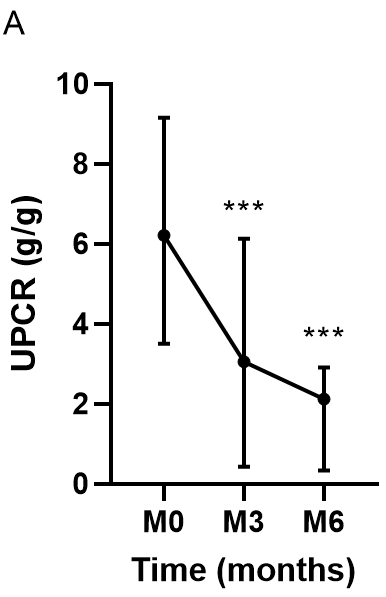

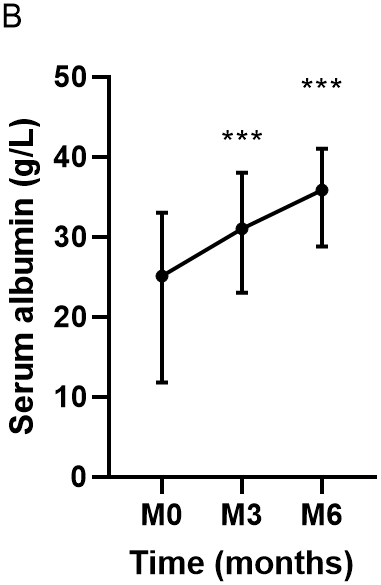

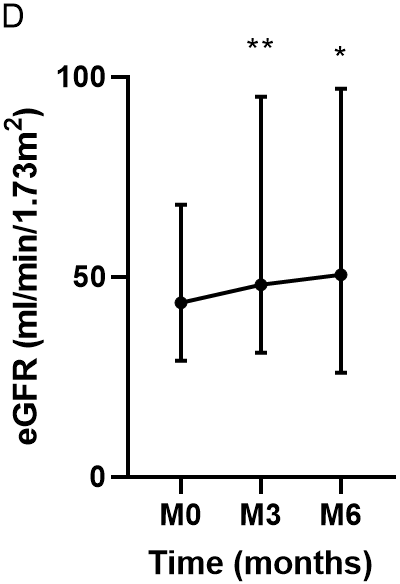

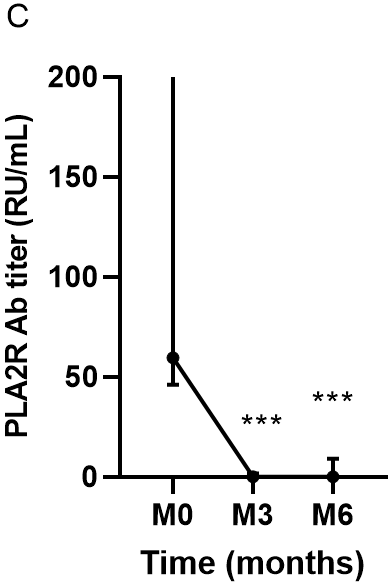
**

Ab: antibody; eGFR: estimated glomerular filtration rate (CKD-EPI formula); M0: month-0; M3: month-3; M6: month-6; PLA2R: phospholipase A2 receptor; UPCR: urine protein-creatinine ratio. The bars show median with interquartile range for each variable. *p < 0.05 vs. baseline; **p ≤ 0.01 vs. baseline; ***p ≤ 0.001 vs. baseline

**Supplementary Figure 2. Comparison of clinical remission rate at month-6 with algorithm-based rituximab treatment *versus* standard treatment according to the risk of underdosing estimated by our algorithm.**

**
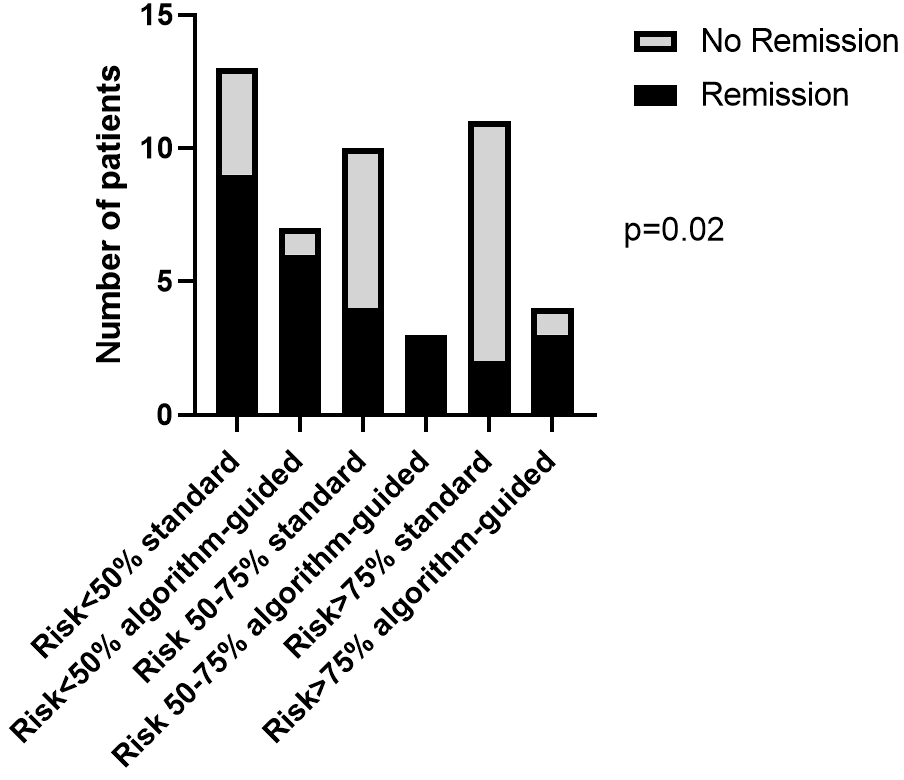
**

Standard treatment: Patients received 1000 mg of rituximab on days 0 and 15 regardless of the risk of underdosing estimated by the algorithm.

Algorithm-guided treatment: Patients with an algorithm-estimated risk of underdosing <50% received 1000mg of rituximab on days 0 and 15. Patients with an estimated risk of underdosing between 50% and 75% received 1000 mg of rituximab on days 0, 15, and 30. Patients with an estimated risk of underdosing >75% received 1000 mg of rituximab on days 0, 15, 30 and 45.

**Supplementary Table 1. Summary of six-month outcomes from the rituximab trials in patients with membranous nephropathy**

| **Trials** | **n** | **Immunological remission^*^** | **Clinical remission** |
| --- | --- | --- | --- |
| **Algorithm-guided rituximab treatment** | 14 | 86 % | 86% |
| GEMRITUX rituximab ^a^ | 37 | 50% | 35% |
| GEMRITUX NIAT ^a^ | 38 | 12% | 21% |
| RI-CYCLO rituximab ^b^ | 37 | 63% | 51% |
| RI-CYCLO cyclophosphamide-corticosteroid ^b^ | 37 | 50% | 65% |
| MENTOR rituximab ^c^ | 65 | 52% | 35% |
| MENTOR cyclosporin ^c^ | 65 | 28% | 49% |
| STARMEN tacrolimus-rituximab ^d^ | 43 | 70% | 44% |
| STARMEN cyclophosphamide-corticosteroid ^d^ | 43 | 92% | 74% |
| High-dose rituximab ^e^ | 28 | 78% | 64% |

NIAT, non-immunosuppressive anti-proteinuric treatment

* Immunological remission was defined by negative PLA2R serology, but the assays used differed between studies: (i) GEMRITUX, STARMEN and high-dose rituximab: anti-PLA2R antibodies were measured by ELISA using a positivity threshold of 14 RU/mL (EUROIMMUN, Lübeck, Germany); (ii) RI-CYCLO: anti-PLA2R antibodies were measured by ELISA using a positivity threshold of 20 RU/mL (EUROIMMUN, Lübeck, Germany); and (iii) MENTOR: another ELISA using a positivity threshold of 40 RU/mL.

^a^ DOI: 10.1681/ASN.2016040449

^b^ DOI: 10.1681/ASN.2020071091

^c^ DOI: 10.1056/NEJMoa1814427

^d^ DOI: 10.1016/j.kint.2020.10.014

^e^ DOI: 10.2215/CJN.11791018
